# Nanoparticle Formulations of Poly (ADP-ribose) Polymerase Inhibitors for Cancer Therapy

**DOI:** 10.3389/fchem.2020.594619

**Published:** 2020-11-23

**Authors:** Bijay Singh, Shicheng Yang, Apurva Krishna, Srinivas Sridhar

**Affiliations:** ^1^Nanomedicine Innovation Center, Northeastern University, Boston, MA, United States; ^2^Department of Physics, Northeastern University, Boston, MA, United States; ^3^Department of Chemical Engineering, Northeastern University, Boston, MA, United States; ^4^Department of Bioengineering, Northeastern University, Boston, MA, United States; ^5^Department of Radiation Oncology, Harvard Medical School, Boston, MA, United States

**Keywords:** Cancer therapy, nanomedicine, nanoparticle, poly(ADP-ribose) polymerase (PARP), PARP inhibitor

## Abstract

A number of poly(ADP-ribose) polymerase (PARP) inhibitors have been recently approved for clinical use in BRCA mutated and other cancers. However, off-target toxicity of PARP inhibitors and the emergence of drug resistance following prolonged administration of these inhibitors indicate the need for improved methods of drug delivery to the tumors. Nanomedicines based upon nanoparticle formulations of conventional small molecule drugs and inhibitors offer many advantages, such as increased solubility and bioavailability of drugs, reduced toxicity and drug resistance, and improved tissue selectivity and therapeutic efficacy. This review highlights the current trends in formulations of PARP inhibitors developed by nanotechnology approaches and provides an insight into the applications and limitations of these PARP inhibitor nanomedicines for cancer therapies.

## Introduction

Although cancer has remained one of the leading causes of death in the US for decades, the 5-year relative survival rate for most cancers has been improving over time (Siegel et al., 2020). The improvement in the survival of patients is largely due to the tremendous progress in cancer biology that enlightens our understanding and produces insights for the diagnosis, prevention, and treatment of cancer. In the last three decades, the US Food and Drug Administration (FDA) has approved more than 100 drugs for anticancer treatment (Blagosklonny, 2004; Kinch, 2014; Sun et al., 2017). Among them, the most prominent class are small molecule inhibitors, which aim at blocking some key receptors or enzymes (Prendergast et al., 2018; Song et al., 2018; Dinavahi et al., 2019), interfere with downstream intracellular signaling molecules (Pantelidou et al., 2019), introduce genetic damage and prevent the DNA repair (Baretti and Le, 2018; D'Andrea, 2018; Jachimowicz et al., 2019), slow down or stop the cell cycle (Cheng et al., 2019; Spring et al., 2019), and eventually lead to the death of cancer cells (Gerber, 2008). These chemotherapy drugs can shrink the solid tumors or slow down their growth, helping patients to live longer with improved quality of life (Gwynne et al., 2017).

A major problem of classical chemotherapy drugs is the emergence of drug resistance leading to diminished response to the drug therapy (Salaroglio et al., 2017; Kopecka et al., 2020). Because most of these drugs directly interfere with the DNA of the cells, any mutation in the DNA empowers the cell to develop resistance against the drugs (Geretto et al., 2017; Faria et al., 2018; Hu et al., 2019). Hence, new anticancer small molecule drugs are being designed to target the proteins responsible for DNA replications in the cancer cells. One of the promising targets is poly(ADP-ribose) polymerase (PARP), a DNA repair protein that plays a key role in nucleotide or base excision repair of DNA damages in cells including DNA breaks instigated by chemotherapy drugs (Morales et al., 2014; Brown et al., 2017). Among several forms of PARP, PARP1 catalyzes a process called PARylation (Schreiber et al., 2006; Zhang et al., 2020) and plays a vital role in regulating chromatin structure as well as enhancing DNA repair (Gibson and Kraus, 2012; Ray Chaudhuri and Nussenzweig, 2017). Normally, PARP1 is upregulated in breast, uterine, ovarian, lung, and skin cancers (Ossovskaya et al., 2010) but is highly expressed in certain cancer types with BRCA1 mutations that display defects in homologous recombination (HR). The BRCA1 protein acts as a tumor suppressor which prevents cancer cells from growing in an uncontrolled way. The BRCA1 protein also plays a critical role in repairing damaged DNA (Savage and Harkin, 2015; Faraoni and Graziani, 2018). Since cancer cells with BRCA1 mutations mostly rely on PARP1 to repair damaged DNA for survival, PARP inhibitors can sensitize the cancer cells inducing synthetic lethality (Tangutoori et al., 2015; Faraoni and Graziani, 2018).

**Figure 1:**
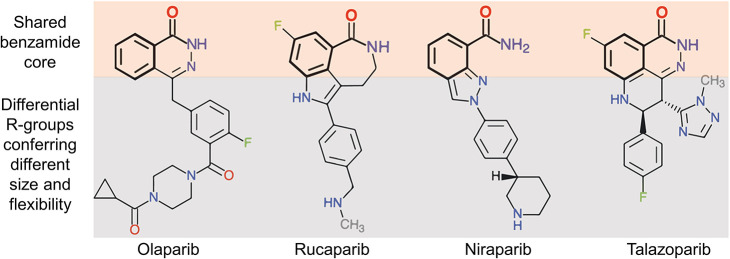
Chemical structures of the four FDA-approved PARP inhibitors. These inhibitors share a common pharmacophore (upper orange shading) while the dissimilar parts of the inhibitors confer them with different size and flexibility (lower gray shading). (Reprinted with permission from Antolin et al., 2020).

Over the past few years, there have been tremendous efforts to develop novel PARP inhibitors for various cancer treatments (Lord and Ashworth, 2017; Mateo et al., 2019). Currently, four PARP inhibitors i.e., olaparib, rucaparib, niraparib, and talazoparib have been approved by FDA for clinical use as monotherapy or maintenance therapy in ovarian and breast cancer with BRCA mutations (Kim et al., 2015; Balasubramaniam et al., 2017; Ison et al., 2018; McCann and Hurvitz, 2018). Interestingly, all four clinical PARP inhibitors share a common benzamide core but they have different side chains in their chemical structures that vary significantly in their mechanism of action and toxicity (Figure 1). Comparatively, these inhibitors differ in their PARP trapping efficiencies (talazoparib > niraparib > rucaparib > olaparib) (Patel et al., 2020) and hence their half-maximal inhibitory concentrations (IC_50_) values follow a reverse pattern (talazoparib < niraparib < rucaparib < olaparib) (Antolin et al., 2020). Current formulations of the small molecule inhibitors are mostly oral tablets mainly due to ease of administration (Jeong et al., 2013). Of note, the amount of PARP inhibitor administered through the oral route is exceedingly high when compared to their IC_50_ values, and this may be attributed as a significant factor in the emergence of PARP inhibitor resistance. Besides, the maximum tolerated dose at oral administration also exposes the body to a range of adverse side effects. To overcome these defects, researchers have been striving for favorable approaches in order to decrease the toxicity of drugs while widening the therapeutic window.

## Nanoparticle-Mediated Drug Delivery

**Figure 2:**
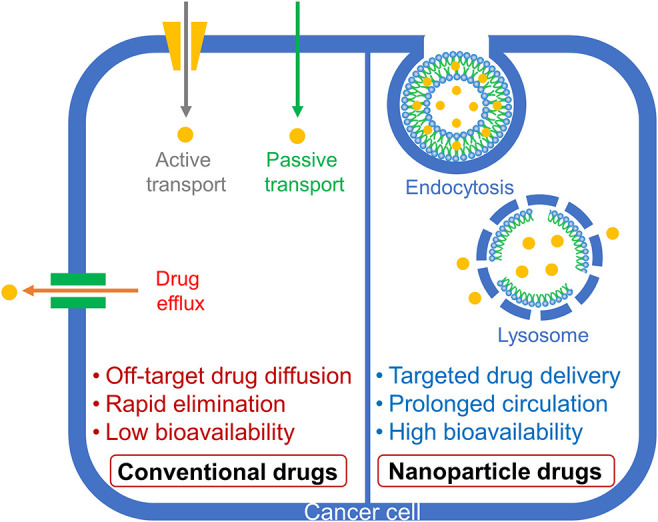
Schematic representation of conventional and nanoparticle drug delivery in cells.

Nanoparticles serve as a unique platform of drug delivery and therefore they have been extensively investigated for their potential use in anti-cancer drug delivery (Amreddy et al., 2018; Riley et al., 2019; White et al., 2019). Nanoparticles can be fabricated in a variety of ways to increase the drug encapsulation capacity at the inner core, and they can be also equipped with multiple functions on the outer core to improve the drug activity in the target environment. Besides, nanoparticles have the potential to deliver poorly water soluble drugs and provide a sustained releasing profile to prolong the blood circulation time (Ventola, 2012; Li et al., 2016; Amreddy et al., 2017; Muhamad et al., 2018). Thus, nanoparticles offer far superior pharmacokinetics compared to small molecule drugs. Many promising drugs fail to pass clinical trials due to their short half-life and high toxicity *in vivo*. Besides, orally administered drugs undergo extensive degradation in the liver resulting in decreased optimum concentration of the drugs before reaching the target site. However, if the drugs could be loaded in specially designed nanoparticles for delivery, the drugs would circulate for longer times in the blood circulation enabling sustained interaction with the tumor and leading to increased tumor accumulation. Nanoparticles also serve as a sheath that would shield off-target toxicities of drugs, alter the cellular uptake of the drugs and lessen the probability of the emergence of drug resistance (Figure 2). At present, many nanoparticles are being studied in clinical trials for a wide variety of medical treatments, and a few nanoparticles have been clinically approved for chemotherapies (Pillai, 2014; Ventola, 2017).

## Formulations of Nanoparticles With PARP Inhibitors

**Figure 3:**
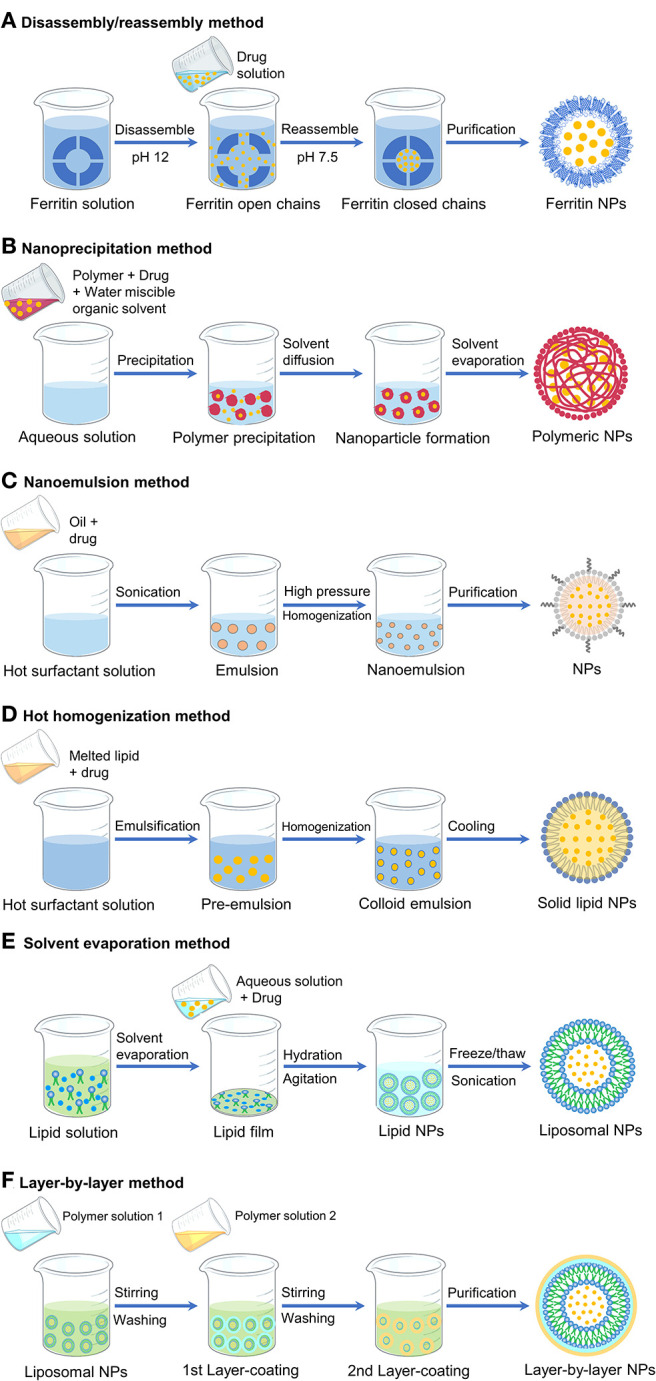
Schematic representation of different formulation methods to produce various nanoparticles (NPs).

Diverse formulations have been developed to yield nanoparticles that varied in compositions, size distributions, and surface properties (Zielińska et al., 2020), all considered as key parameters while designing nanoparticles for safe delivery. Here, we explore the methods available for the preparation of clinically approved PARP inhibitors in various nanoparticle forms (Figure 3). Since these inhibitors in free form are poorly water soluble, emulsion and precipitation are the two suitable methods to formulate the nanoparticles of PARP inhibitors. In either method, the development process ensures the incorporation and stabilization of drugs inside the nanoparticles while making colloidal formulations. A list of nanoparticles of PARP inhibitors in the literatures is summarized in Table 1.

**Table 1:** A list of nanoparticles of PARP inhibitors and their uses.

**Formulation**	**Drug**	**Size (nm)**	**ZP (mV)**	***In vitro***	***In vivo***	**Route**	**Reference**
Nanoparticles	Olaparib	35	−38 ± 5	Breast cancer	NA	NA	Caster et al., 2015
Nanoparticles	Olaparib	14.3 ± 4.2	−20.5 ± 1.2	BRCA-mutated TNBC	NA	NA	Mazzucchelli et al., 2017
Nanoparticles	Olaparib	71 ± 5	−24 ± 7	Radiation-resistant prostate cancer	Xenograft mouse model of prostate cancer	IV	van de Ven et al., 2017
Nanoparticles	Olaparib	72.8 ± 5.8	−30.5 ± 9.0	Ovarian cancer	Disseminated peritoneal disease model	IP	Baldwin et al., 2018
Nanoparticles	Olaparib, Gemcitabine	131.2 ± 3.1	NA	BRCA2-mutant pancreatic cancer	Xenograft mouse model pancreatic cancer	IV	Du et al., 2018
Nanoparticles	Talazoparib	71.4 ± 12.0	3.98 ± 2.3	BRCA mutant fallopian tube	Intraperitoneal disseminated disease model	IP	Baldwin et al., 2019b
Nanoparticles	Talazoparib	74.5 ± 11.0	15.3 ± 1.6	BRCA-mutated breast cancer	Xenograft mouse model of human breast cancer	IV	Zhang et al., 2019
Nanoemulsion	Talazoparib	151.4 ± 0.7	−33.3 ± 1.2	BRCA1-mutant ovarian cancer, TNBC	NA	NA	Mehra et al., 2018
Nanoemulsion	Olaparib	144–157	−6.03	Small cell lung cancer (SCLC)	Xenograft mouse model of SCLC	IV	Gonzales et al., 2018
Nanocapsules	Olaparib	107 ± 6	−38	BRCA1/2 wild breast cancer	NA	NA	Novohradsky et al., 2018
Solid lipid nanoparticles	Talazoparib	218	−28.5	BRCA1 mutant TNBC	NA	NA	Guney Eskiler et al., 2018
Lipospheres	Olaparib	126.7 ± 4.5	NA	NA	Sprague Dawley rats	Oral	Pathade et al., 2019
Layer-by-layer nanoparticles	Olaparib, Talazoparib	100 ± 12	−31 ± 6	Ovarian cancer	Xenograft mouse model of ovarian cancer	IV	Mensah et al., 2019

*ZP, Zeta potential; TNBC, Triple negative breast cancer; IV, Intravenous; IP, Intraperitoneal; NA, Not available*.

### Disassembly/Reassembly Method

Olaparib loaded in human ferritin nanoparticles (HFn) were produced by disassembly/reassembly method (Mazzucchelli et al., 2017). Ferritins are iron storage proteins consisting of self-assembling 24 subunits to form a spherical cage architecture with a size of 12 nm (Bellini et al., 2014). These subunits can be disassembled at acidic or basic pH and reassembled to nanocage architecture again by changing the pH to neutrality (Kim et al., 2011). To load drugs, HFn (2 mg), produced by recombinant DNA technology in *E. coli*, was disassembled in 0.15 M NaCl solution (1.5 mL) by adding 0.1 M NaOH solution while maintaining the pH 12. Secondly, olaparib powder (0.5 mg) was dissolved in 0.1 M NaOH (0.5 mL) by sonication and incubated with the disassembled HFn solution for 10 min at room temperature. The resulting solution was brought to pH 7.5 using 0.1 M HCl and stirred for 2 h allowing to reassemble the HFn with olaparib (HOla). The HOla nanoparticles were concentrated using a 100 kDa Amicon filter and purified using a spin desalting column to remove unloaded olaparib.

Advantages: Ferritins are natural protein nanoparticles commonly found in animals, plants, and microorganisms. Ferritins form a unique spherical shell-like architecture of nanosize that provides two interfaces, an outer layer for possible functional loading and an inner cavity for drug loading. The protein architecture can be distorted in an acidic condition and restored at the physiological condition. In addition, ferritins are stable at high temperatures (75°C) and various denaturants. With these unique characteristics, ferritin nanoparticles have found broad applications in drug delivery, vaccine development, bioassay, and molecular imaging (Wang et al., 2017).

Disadvantages: The method of producing ferritin nanoparticles in microorganisms requires tedious purification steps. As a result, mass production of ferritin nanoparticles is a difficult task. Besides, the small size of ferritin nanoparticles limits the high drug loading capacity. Ferritin nanoparticles still face challenges for clinical translation due to insubstantial data on biocompatibility and toxicity of various ferritins.

### Nanoprecipitation Method

Lipid/polymer hybrid nanoparticles of olaparib were formulated through a single step nanoprecipitation method (Zhang et al., 2008; Valencia et al., 2010; Caster et al., 2015; Baldwin et al., 2016). Briefly, a lipid solution was prepared by mixing 1,2-distearoyl-sn-glycero-3 phosphoethanolamine-N-methoxy(poly-ethyleneglycol)(DSPE-PEG)-COOH (1 mg/ml) and lecithin (1 mg/ml), each dissolved in 4% ethanol aqueous solution, in the molar ratio of 7:3. The lipid solution was heated at 65°C for 15 min with stirring. To formulate the drug loaded nanoparticles, 100 μL of PLGA (10 mg/ml) and 100 μL of olaparib (3.0 mg/ml) in acetone solutions were mixed in 800 μL of acetonitrile and added dropwise to the above heated lipid solution. Soon after, the mixture was vortexed and stirred at room temperature for 1 h allowing the nanoparticles to self-assemble. The nanoparticles were washed using dialysis filters and suspended in PBS for further experiments.

Nanoformulation of talazoparib was produced using NanoAssemblr Benchtop through a controlled nanoprecipitation method. NanoAssemblr platform produces nanoparticles through a controlled nanoprecipitation method in which the lipids dissolved in a low-polarity organic solvent are mixed with aqueous buffer leading to the spontaneous self-assembly of the lipids into unilamellar vesicles of nanometer size. Nanoformulation of talazoparib was synthesized using the lipid core of 1, 2-dipalmitoyl-sn-glycero-3-phosphocholine (DPPC), DSPE-PEG, 1,2-dioleoyl-3-trimethyl-ammonium-propane (DOTAP) and cholesterol to encapsulate talazoparib (Zhang et al., 2019). The lipid core components were separately dissolved in ethanol and mixed them together with talazoparib solution. The lipid/drug mixture was rapidly precipitated with PBS through the microfluidic mixing chamber using NanoAssemblr Benchtop. The organic solvents in the precipitated formulation were purged with inert argon and dialyzed against phosphate buffered saline to produce the nanoformulation of talazoparib.

Advantages: The formation of nanoparticles via nanoprecipitation method occurs by spontaneous diffusion of an organic solution into an aqueous solution. Therefore, the method does not need mixing devices that require high amount of pressure, energy, or shear. Besides, the flow rate and ratio of this method can be controlled to adjust the sizes of nanoparticles. It is easy to scale up the final product by simply increasing the total volume of aqueous and organic solutions. No multiple batches of nanoparticle synthesis reactions are required.

Disadvantages: A major drawback of nanoprecipitation method is that the choice of materials for the preparation of nanoparticles is restricted only to selective polymers and solvents. Another drawback is that organic solvents remain in the final products even after the evaporation step, which is a major safety concern of these formulations to enter clinical trials.

### Nanoemulsion Method

Nanoemulsion of talazoparib was prepared by high pressure homogenization system (Mehra et al., 2018). The aqueous phase was made by a continuous stirring of Tween 80 (4%), glycerin (2.2%), sorbic acid (0.1%), sodium acetate (0.05%), boric acid (0.1%), and sodium EDTA (0.02%), all components dissolved in deionized water in w/v ratio, at 70°C. The oil phase was made by dissolving talazoparib (5 mg) in trans-caryophyllene (β-CP) (400 mg) and olive oil (100 mg) via sonication. The oil phase was continuously dropped into the aqueous phase while stirring at 13,000 rpm for 1 h at room temperature to make a coarse emulsion which was further subjected to high pressure emulsification to obtain the final emulsion. In this way, nanoemulsion with or without drugs could be prepared.

Nanoemulsion loaded with fluorescently labeled olaparib (Ola-FL) was prepared by the following steps (Gonzales et al., 2018). A lipid solution was made using 1,2-distearoyl-sn-glycero-3-phosphocholine (DSPC), cholesterol and DSPE-PEG (62:33:5 mole ratio) in ethanol to make a concentration of 25 mg/ml. The lipid solution was first mixed with a 150 μL of Ola-FL (2.26 mM) and Miglyol 812 N, injected into 20 mL of PBS and stirred at 1,400 rpm for 13 min. The resulting nanoemulsion was centrifuged at 4,000 rpm at 22°C for 30 min to get rid of aggregates, washed with PBS through centrifugal concentrators using 100 kDa molecular weight cut-off, and concentrated by Tangential Flow Filtration System. The formulation was finally sterilized through a PES syringe filter (0.22 μm).

Advantages: Nanoemulsions can be formulated in variety of formulations such as sprays, creams, and liquids (Jaiswal et al., 2015) to administer drugs through various routes like oral, topical and intravenous (Tayeb and Sainsbury, 2018). Nanoemulsions can be made with both hydrophilic and lipophilic drugs. In fact, nanoemulsions often enhance higher loading capacity for lipophilic drugs, and increase bioavailability of the drugs. The other advantage of nanoemulsion method is the capability of large-scale production by high-pressure homogenization.

Disadvantages: A major concern of nanoemulsions for pharmaceutical applications is their stability compared to other nanoformulations. To avoid the decomposition of nanoemulsions from unfavorable conditions such as temperature change and mechanical forces, addition of suitable stabilizers may be necessary to improve the physical stability of nanoemulsions (Liu et al., 2019).

### Hot Homogenization Method

Solid lipid nanoparticles of talazoparib formulation were prepared by a hot homogenization method (Guney Eskiler et al., 2018). Initially, 2.5% glycerol monostearate was melted at 80°C and talazoparib was homogeneously dispersed into the melted lipid. The drug containing melted lipid was then emulsified under stirring in a hot aqueous surfactant solution of 2.0% Tween 80 heated at 80°C. The pre-emulsion thus obtained was homogenized using a high-speed homogenizer at 20,500 rpm for 10 min. The produced hot nanoemulsion was cooled down to room temperature until the lipid recrystallized and formed solid lipid nanoparticles.

Lipospheres of olaparib were also prepared and optimized through this method (Pathade et al., 2019). The formulation of lipospheres was prepared using egg lecithin as coat lipid, triglycerides as core lipids, and Tween 80, Span 80, and Cremophor RH 40 as surfactants. The lipospheres were optimized by varying the above components, including kind of core lipid, solvent type, ratio of core lipid to coat lipid and ratio of total lipid to total surfactant. Typically, an optimized method was proceeded by dissolving 13 mg of egg lecithin in an appropriate amount of N-methyl pyrrolidone at 50°C. The egg lecithin solution was homogeneously dispersed into a melted solution of the surfactants (each 35 mg) at 60°C with continuous stirring to form pre-concentrate. Finally, olaparib was blended with the pre-concentrate by vortexing for 15 min.

Advantages: Compared to other methods, the key advantage of hot homogenization method is that it proceeds without the use of toxic organic solvent. The formulation thus produced is regarded as safe easing for the approval of clinical translation. The method is easy to scale-up and it can incorporate both hydrophilic and lipophilic drugs (Ghasemiyeh and Mohammadi-Samani, 2018).

Disadvantages: Despite the above-mentioned advantages, the method is not appropriate for heat-sensitive drugs, and it does not produce a narrow particle size distribution (Naseri et al., 2015). Other disadvantages of solid lipid nanoparticles are gelation tendency and low drug loading efficiency because of their crystalline structure (Yoon et al., 2013). High amount of surfactant will be considered as a disadvantage.

### Solvent Evaporation Method

Nanoparticles of olaparib were made using DPPC, cholesterol, DSPE-PEG, DOTAP, and olaparib (van de Ven et al., 2017). The above components were individually dissolved in chloroform, mixed at a defined molar ratio, and dried under a rotary vacuum overnight to evaporate the solvent. The dried lipid-drug residues were hydrated using PBS (pH 7.4), emulsified by repeated heating and cooling with agitation, and then sonicated at 25°C for 10 min to produce lipid nanoparticles of olaparib.

Liposomal nanoparticles of olaparib were prepared by a standard procedure of hydrating dry lipid films (Novohradsky et al., 2018). First, a film consisting of a mixture of 1,2-dioleoyl-sn-glycero-3-phosphocholine, 1,2-dioleoyl-sn-glycero-3-phosphoserine, 1,2-distearoyl-sn-glycero-3-phosphoethanolamine-N-[amino(polyethylene glycol)-2000] and cholesterol was made by a solvent evaporation method. The lyophilized phospholipid film was hydrated by a solution of olaparib and/or carboplatin while incubating at 37°C for 60 min, followed by freeze-thaw cycles using a dry-ice/ethanol bath (−70°C) and a water bath (37°C). The resulting suspension was centrifuged at 15,000 rpm for 10 min at 4°C to collect the nanocapsules in the pellet and washed with water several times to remove the impurities.

Advantages: The method is relatively simple for mass production and modification of drug encapsulation and release (Piñón-Segundo et al., 2012). Organic solvent is evaporated before hydration that decreases the potential residues with the nanoparticles prepared in this method.

Disadvantages: Organic residues may remain intact with the nanoparticles due to incomplete solvent removal. The other disadvantage of this method is that there is no way to precisely control the particle sizes and shapes.

### Layer-By-Layer Method

Polymeric liposomal nanoparticles were prepared by layer-by-layer method (Mensah et al., 2019). First, Liposomes were prepared by dissolving DSPC, 1-palmitoyl-2-oleoyl-sn-glycero-3-phospho-(10-rac-glycerol) (POPG) and cholesterol in chloroform and methanol mixture at a mass ratio of 56:39:5. ratio together with talazoparib or olaparib. The lipids and drug mixture were evaporated using rotary evaporation at 40°C overnight to form a thin film. The film was later hydrated with citric acid buffer (pH 4.0) in a water bath at 65°C for 1 h under sonication to form the liposomes with drugs. The liposomes were further coated with poly-L-lysine (PLL) and hyaluronic acid (HA) by the layer-by-layer process. The liposomes suspension (1 mg/ml) was added dropwise to a rapidly stirring 45 ml solution of PLL (500 μM). Following the purification, the concentrated PLL-layered liposomes (1 mg/ml) were added dropwise to a 45 mL solution of HA (10 μM) with rapid stirring. The HA-coated-PLL-layered liposomes were recovered by purification.

Advantages: This method prepares liposomes similar to the solvent evaporation method. The difference is that the liposomes are further coated with more layers on the surface. More features can be brought to these nanoparticles by using different kinds of coating materials. For example, the sizes of liposomes can be varied, and their solubility can be modified by surface coatings.

Disadvantages: This method requires multiple steps and is thus expensive. The multilayered nanoparticles have low drug loading capacity and release (Oh et al., 2013).

## Applications of Nanoparticles of PARP Inhibitors

One of the initial reports of lipid-polymer hybrid nanoparticle formulation commenced a robust drug delivery platform in the field of nanomedicine (Zhang et al., 2008). The nanoparticle had high drug encapsulation efficiency, tunable drug releasing capacity, remarkable serum stability, and differential targeting potency. Accordingly, many varieties of nanomedicines have been formulated in an attempt to eliminate the drawbacks of conventional drug delivery systems for anticancer therapies. Although conventional PARP inhibitors seem a promising strategy for the treatment of triple-negative breast cancer (TNBC) with BRCA mutations, small molecule drugs showed no merits in wild type BRCA cancers possibly due to low drug bioavailability and inefficient drug delivery in the nucleus. To overcome these hurdles, ferritin nanoparticles with olaparib (HOla) were developed which are highly stable at 37°C in PBS buffer releasing <3% drugs even after 6 h incubation. When tested on TNBC cells with or without BRCA mutation (Mazzucchelli et al., 2017), HOla nanoparticles induced fast internalization into the cells and enhanced PARP1 cleavage indicating clear evidence of olaparib delivery into the nuclear compartment. Remarkably, HOla nanoparticles displayed significant cytotoxicity in cancer cells demonstrating 1,000-fold higher anticancer activity than free olaparib.

Recently, a strategy of administrating PARP inhibitors with other chemotherapy drugs to increase the greater therapeutic effect on the tumors is being pursued. However, dose-limiting toxicities are major obstacles for this combination therapy. The other challenging aspect of this method is to deliver two drugs concurrently to the tumor site at a proper concentration. To address these issues, an epidermal growth factor receptor targeting GE11 peptide nanoparticle (GENP) was formulated with two drugs (olaparib and gemcitabine) for co-delivery (Du et al., 2018). The resulting nanoparticles, GENP-Gem-Ola could coordinately release both drugs and showed strong synergistic effects *in vitro*. GENP-Gem-Ola also prolonged the half-lives of both drugs *in vivo* and enhanced the tumor accumulation of the drugs at the optimum therapeutic proportion leading to drastic suppression of the tumor growth in a murine model of BRCA-mutant pancreatic cancer. Novel electrostatic layer-by-layer liposomal nanoparticles were designed with HA coating layer to facilitate CD44 receptor targeting of advanced staged high-grade serous ovarian cancer (HGSOC) (Mensah et al., 2019). HA-coated-PLL-layered liposomes were formulated to encapsulate both cisplatin (64%) and olaparib (26%) or talazoparib (21%). Codelivery of encapsulated cisplatin and PARP inhibitor significantly reduced tumor growth in orthotopic model of HGSOC xenografts.

Mehra et al. formulated talazoparib nanoemulsion that contains spherical particles with a polydispersity index (PDI) of 0.120 ± 0.010 and drug encapsulation efficiency of 0.05 % (Mehra et al., 2018). *In vitro* release profile showed a slow release of talazoparib from the nanoemulsion. The percentage of drug released in PBS was 50.4 ± 1.64, 47.7 ± 3.09 and 51.45 ± 2.31 at pH 5.3, 6.5, and 7.4, respectively, in 24 h. Cytotoxicity of talazoparib nanoemulsion was evaluated in breast cancer cells (MDA-MB-231) and ovarian cancer cells (SKOV-3). The IC_50_s of talazoparib nanoemulsion were 0.48 and 1.35 for MDA-MB-231 after 48 and 72 h treatment, respectively. For SKOV-3, the IC_50_s were 11.75 and 0.46 at 48 and 72 h incubation, respectively, indicating the concentration dependent cytotoxicity of the nanoemulsion to the cells. The cellular uptake of coumarin-6 loaded nanoemulsion was higher than free coumarin-6 in MDA-MB-231 suggesting that the nanoemulsion platform would be more appropriate for systemic delivery of the drug.

The clinical potential of many small molecule drugs is limited by their rapid pharmacokinetics which affects the overall pharmacodynamics. To solve the problem, the small molecule drugs are encapsulated into nanoparticles to ameliorate the half-life of the drugs escalating their concentration at the site of action and the resulting effect. As a proof of concept, Gonzales and authors encapsulated a fluorescently labeled olaparib (Ola-FL) by nanoemulsion method to form nanoparticles (Gonzales et al., 2018). To validate the pharmacological performance of Ola-FL *in vivo*, it was intravenously injected in subcutaneous xenograft mice models of human small cell lung cancer (SCLC) (H-69 and H-82). The half-life of the Ola-FL in blood was 6 h while the non-encapsulated fluorescent molecule took only 24.5 min in the body to be reduced by half. Biodistribution and histology studies of SCLC xenografts suggested that the nanoemulsion could specifically deliver Ola-FL to the tumor cell nuclei. Thus, Ola-FL demonstrated a new potential nanoparticulate platform for theranostics that could use not only for therapy but also for imaging of PARP action *in vivo*.

While PARP inhibitors such as talazoparib are currently being used in clinics in patients with BRCA mutation (HR defective) breast and ovarian cancers, the inherent and acquired drug resistance to PARP inhibitors by the restoration of BRCA1/2 function (HR active) is the major challenge to successful therapy. To overcome HR-mediated resistance, Guney Eskiler et al. produced talazoparib loaded solid lipid nanoparticles (SLNs) and they treated 10 nM of talazoparib with or without SLNs in the TNBC cell lines, HCC1937 (talazoparib-sensitive) and HCC1937-R (talazoparib-resistant) for 12 days (Guney Eskiler et al., 2018). The cell viability of HCC1937 reduced to 33.5 ± 1.4% and 29.0 ± 1.3 with talazoparib and talazoparib-SLNs, respectively. Surprisingly, the cell viability of HCC1937-R was nearly 100% and 34.3 ± 2.3% with talazoparib and talazoparib-SLNs, respectively. The results showed that there was no effect of talazoparib in HCC1937-R cells after treatment while talazoparib-SLNs had the potential to overcome talazoparib resistance in HCC1937-R cells. Additionally, they treated 10 nM of talazoparib with or without SLNs in normal human mammary breast epithelial cell line (MCF-10A) for 12 days to analyze the toxicity of the SLNs. The cell viability of MCF-10A cells treated with talazoparib and talazoparib-SLNs were 39.3 ± 1.5 and 63.5 ± 0.8%, indicating that SLNs are safe carriers for cancer drug delivery.

Another challenge of oral administrated PARP inhibitor drug is low oral bioavailability requiring frequent or higher doses of the drug which increases the risk of hematologic toxicity in the patients. To improve the oral bioavailability, Pathade et al. formulated olaparib loaded lipospheres by melt dispersion method (Pathade et al., 2019). This method was well suited to produce an optimum size of nanoparticles as particle size would influence on solubility, dissolution rate and oral bioavailability of the drug (Sun et al., 2012). The loading of olaparib in lipospheres could be varied from 2.5 to 10% (w/w) with entrapment efficiency (100%) at 2.5% drug loading. The lipospheres had 50% cumulative drug release before 1 h and 70.06 ± 1.79% within 10 h. The oral bioavailability of olaparib was investigated in Sprague Dawley rats following a single dose of oral administration of lipospheres. While the half-life of olaparib lipospheres was increased by 1.26-fold in comparison with olaparib alone, the lipospheres reduced hematologic toxicities too.

**Figure 4:**
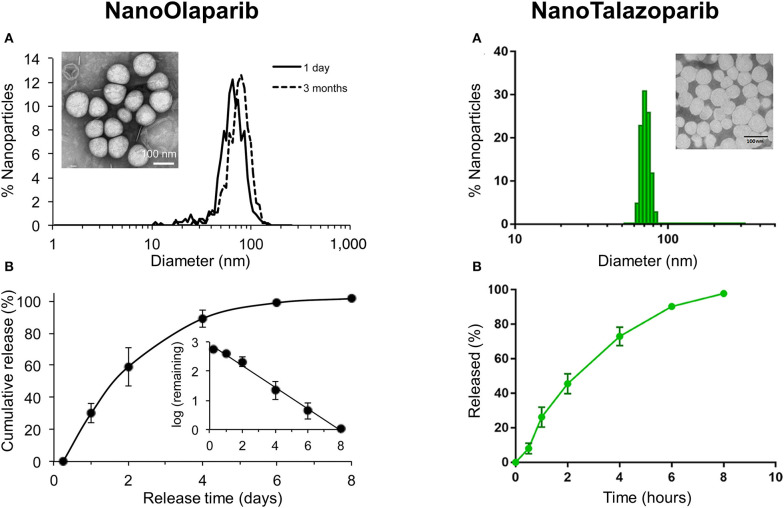
Characterization of NanoOlaparib and NanoTalazoparib. **(A)** Nanoparticle size distribution measured by dynamic light scattering and transmission electron micrograph (TEM) of nanoparticles (Inset). **(B)** Cumulative drug release in phosphate buffered saline at pH 7.4 at 37°C. (Reprinted with permission from van de Ven et al., 2017; Baldwin et al., 2019b).

Initially, Baldwin et al. formulated NanoOlaparib that consists of 5 mg Olaparib/mL of formulation (Baldwin et al., 2018). Under static conditions, NanoOlaparib showed a first-order drug release profile with 100% drug release within 8 days (Figure 4). The commercial oral Olaparib formulation shows 90% drug release within 4 days. The authors also developed an assay to determine the IC_50_ of the PARP inhibitor nanoparticles in cell lines that would help to estimate further dosing for *in vivo* experiments (Baldwin et al., 2017). The assay established that NanoOlaparib was as effective as free Olaparib when tested in four different ovarian cancer cell lines. For *in vivo* experiment, NanoOlaparib was administered daily through intraperitoneal (IP) injection in disseminated peritoneal tumor animal models (Baldwin et al., 2018). Consistent with *in vitro* results, NanoOlaparib seemed to offer improved efficacy than oral Olaparib. However, NanoOlaparib could enter systemic circulation within an hour of administration and showed significant toxicity when given as a daily dosing regimen resulting in the premature death of tumor models. Decreasing the doses of NanoOlaparib administration twice a week led to no toxicity but also no therapeutic efficacy.

In order to address this issue, Baldwin et al. developed NanoTalazoparib that had a drug encapsulation efficiency of 76.9 ± 11.35% with drug loading content of therapeutic concentration of talazoparib (153.8 ± 22.7 μg/mL) (Baldwin et al., 2019b). Of note, a panel of murine and human BRCA cell lines was more sensitive to NanoTalazoparib showing a 10-fold decrease in IC_50_ value compared to NanoOlaparib. Further, therapeutic efficacy was tested in an intraperitoneal murine disseminated disease model. By administering three doses weekly by intraperitoneal injection, NanoTalazoparib retarded disease progression and significantly reduced the formation of ascites. In another study, a combination of talazoparib with temozolomide has shown to be greatly effective against Ewing sarcoma (Smith et al., 2015). However, this combination was toxic imposing a reduction of TMZ dose for suitable outcomes. Unlike talazoparib, NanoTalazoparib showed low toxicity and prolonged half-life of drugs contributing to a greater response rate with temozolomide in TC-71 Ewing sarcoma xenografts (Baldwin et al., 2019a).

Additionally, nanoparticle PARP inhibitors have shown promising results in combination with radiotherapy to enhance local DNA damage in tumors. NanoOlaparib pretreatment enhanced DNA damage and demonstrated increased sensitivity to radiation in three radiation-resistant prostate cancer cell lines (LNCaP, PC-3, and FKO1) (van de Ven et al., 2017). Irradiation in mice subcutaneously implanted with FKO1 cells following intravenous (IV) administration of NanoOlaparib resulted in significant inhibition of tumor growth than radiation alone.

## Summary and Outlook

PARP inhibitors have shown potent activity as monotherapy or in combination with other drugs or inhibitors to treat BRCA mutated cancers in both preclinical and clinical settings. However, the defects associated with small molecule drugs such as generating drug resistance and off-target toxicity also exist in PARP inhibitors. To address these issues, PARP inhibitors are formulated into nanomedicines which could alter the drug uptake pathways potentially overcoming the emergence of drug resistance and minimizing the direct contact of drugs with healthy tissues reducing the drug toxicity. Here, we have reviewed several nanotechnology approaches to produce nanomedicines of PARP inhibitors and their therapeutic uses in different cancers including breast, ovarian, pancreatic, lung, and prostate. In most of these treatments, nanomedicines of PARP inhibitors have shown superiority over the PARP inhibitors as single agents. In contrast to oral formulations of PARP inhibitors, nanomedicines are administered through IP or IV injections and these administration routes increase the bioavailability of the drugs with required doses. The other advantages of nanomedicine are longer blood circulation time and reduced off-target toxicity due to increased tumor uptake. Because cancer patients vary case by case, it is also imperative to develop personalized therapy by identifying reliable biomarkers on the tumor of the patients for accurate treatment with the suitable PARP inhibitors. PARP inhibitors could also be developed as imaging agents that render safe, targeted and efficient diagnosis and therapy together for individual patients by offering the “proper” drug at the “proper” dose according to the test results (Jeelani et al., 2014).

Based on the concept that two are better than one, combination therapies of PARP inhibitors with radiation or other chemotherapy drugs would require lower doses of drugs thus providing safer and tolerable treatment for cancer patients. Besides, combination therapy works in two modes of action, for which numerous studies have demonstrated stronger evidence of enhanced therapeutic benefits. The effectiveness of combination therapies raises hopes of more efficient treatments and hence several PARP inhibitors are being investigated to assess their therapeutic efficacies in non-BRCA cancers too. A combined nanoparticle drug conjugate with olaparib (NCT02769962) and a liposomal combination of drugs including rucaparib (NCT03337087) are under clinical trials now (ClinicalTrials.gov). However, nanomedicines still face challenges, such as the need for better characterization of the formulations, storage problems, manufacturing costs, and consumer compliance. With the advancement of nanotechnologies and the better understanding of the molecular mechanisms of nanomedicine actions, these developments will create improved treatment methods to control and prevent the progress, spread or return of cancer.

## Author Contributions

All authors have made a substantial, direct and intellectual contribution in writing, editing, and reviewing the manuscript for publication.

## Conflict of Interest

The authors declare that the research was conducted in the absence of any commercial or financial relationships that could be construed as a potential conflict of interest.
